# Synthetic Modulators of the Vitamin D Receptor: From Structural Innovation to Disease-Specific Applications

**DOI:** 10.3390/biom16030396

**Published:** 2026-03-06

**Authors:** Tram Thi-Ngoc Nguyen, Tomohiro Kurokawa, Yoshiaki Kanemoto, Takahiro Sawada, Shigeaki Kato

**Affiliations:** 1 Department of Pharmacy, Iryo Sosei University, Iino, Chuo-dai, Iwaki 970-8551, Fukushima, Japan; 2 Research Institute of Innovative Medicine, Tokiwa Foundation, Iwaki 971-8112, Fukushima, Japan; 3 School of Medicine, Fukushima Medical University, Fukushima 960-1295, Fukushima, Japan

**Keywords:** Vitamin D Receptor (VDR), synthetic vitamin D analogs, selective nuclear receptor modulators, ligand-dependent transcription, helix-12 conformational change, hypercalcemia

## Abstract

Vitamin D signaling via the vitamin D receptor (VDR) regulates calcium–phosphate homeostasis and extensive gene programs controlling cell proliferation, differentiation, immune tone, and metabolism. However, systemic use of the natural agonist 1α,25-dihydroxyvitamin D_3_ (calcitriol) for extraskeletal indications is limited by dose-limiting hypercalcemia. This review summarizes VDR biology and the structural basis of ligand action, emphasizing how ligand-induced repositioning of helix 12 and altered coregulator recruitment can be exploited to engineer selective VDR modulators. We highlight medicinal chemistry strategies spanning secosteroidal analogs with side-chain or ring modifications and emerging non-seco scaffolds and discuss clinically established agents (e.g., calcipotriol and paricalcitol) alongside experimental “super-agonists”, partial agonists, and antagonists designed to widen the therapeutic window. Finally, we discuss current evidence for VDR targeting across cancer, metabolic disease, fibrosis, and immune-inflammatory disorders, including mechanisms of resistance such as dysregulated vitamin D metabolism and epigenetic repression. Structural and epigenomic insights are positioning next-generation VDR ligands as tissue- and pathway-biased therapeutics that may enable safer, mechanism-guided translation beyond bone and mineral indications.

## 1. Introduction

Vitamin D, traditionally recognized for its critical role in calcium and phosphate homeostasis, has emerged as a multifunctional secosteroid hormone influencing diverse biological systems far beyond skeletal health [1,2,3]. The hormonally active form, 1α,25-dihydroxyvitamin D_3_, exerts its effects primarily through the vitamin D receptor (VDR)—a ligand-activated nuclear transcription factor belonging to the steroid/thyroid hormone receptor superfamily [4,5,6]. Upon ligand binding, VDR heterodimerizes with the retinoid X receptor (RXR) and regulates transcription of hundreds of genes via vitamin D response-elements (VDREs) distributed throughout the genome [7,8,9]. This network controls not only mineral metabolism but also processes such as cell proliferation, differentiation, immune modulation, and metabolic homeostasis, thereby linking vitamin D signaling to the pathophysiology of cancer, fibrosis, autoimmune and metabolic diseases [1,3,6].

The pleiotropic nature of VDR signaling has positioned it as an attractive therapeutic target. However, clinical translation of natural vitamin D metabolites—particularly calcitriol—has been hampered by dose-limiting hypercalcemia, a consequence of its potent effects on calcium absorption and bone resorption [4,6]. To circumvent this obstacle and selectively exploit the non-classical actions of VDR, intense medicinal chemistry efforts over the past decades have focused on designing synthetic VDR ligands (vitamin D analogs and non-secosteroidal modulators). These compounds aim to retain or even enhance the beneficial gene-regulatory and antiproliferative effects of 1,25(OH)_2_D_3_ while minimizing its calcemic liabilities [4,10].

Beyond structural innovation, advances in VDR crystallography and molecular docking have enabled rational design of ligands that bias receptor conformation and coregulator recruitment, giving rise to selective VDR modulators (SVDRMs) analogous to selective estrogen receptor modulators (SERMs) [4,7]. These efforts have produced clinically used analogs such as calcipotriol (for psoriasis) and paricalcitol (for secondary hyperparathyroidism), as well as experimental “super-agonists” like inecalcitol and tissue-specific agents showing promise in oncology, metabolic, and fibrotic disorders [10,11].

Contemporary research has expanded the functional landscape of VDR ligands from bone-centric pharmacology toward systemic and cell-type-specific interventions. Synthetic VDR agonists and antagonists are now being explored for anticancer therapy, modulation of inflammatory and immune pathways, and attenuation of organ fibrosis [6,11]. Moreover, understanding of VDR’s epigenomic and non-genomic mechanisms—including its interactions with chromatin modifiers, microRNAs, and intracellular signaling cascades—has opened new therapeutic perspectives [6,8]. In this short review, we discuss not only the biological actions of the vitamin D receptor (VDR) and its natural ligand, but also the strategies used to design and synthesize novel VDR ligands. Particular attention is given to recent structural innovations in synthetic analogs that aim to enhance receptor selectivity, potency, and therapeutic efficacy.

## 2. VDR Biology and Natural Ligand

**Figure 1:**
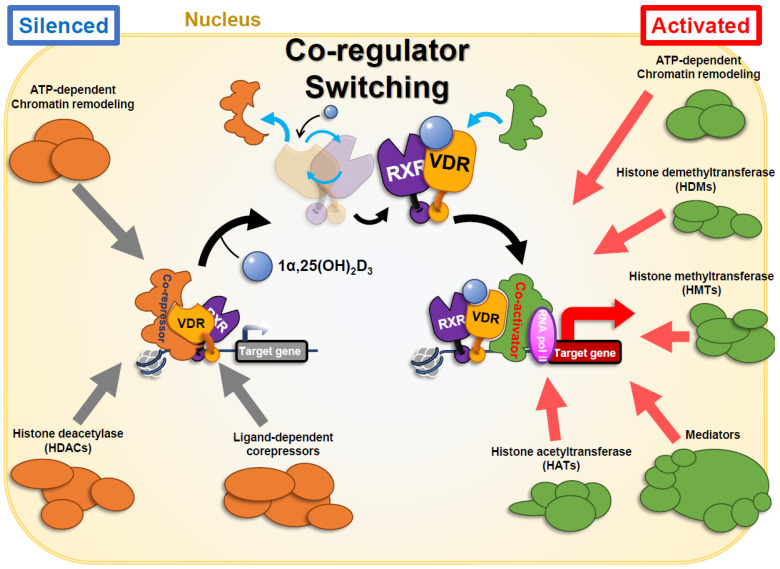
A schematic view of ligand-dependent transactivation of VDR via coregulator switching. VDR is transcriptionally silent in the absence of an agonist even when bound to its target. DNA elements on chromatin in the VDR target genes induced by VDR agonists [5,9]. Repressive function of VDR is considered owing to its association with transcriptional corepressors. Ligand binding to VDR triggers shifting of the C-terminal of α-helix 12, leading dynamic alteration of the whole VDR protein structure and dissociating the corepressors [19,23,24]. Instead, transcriptional coactivators are recruited to VDR to transactivate gene expression [25,26]. Transcriptional coregulators are diverse and associated with VDR dependent on the environment of the promoters of the VDR target genes. Rapid gene regulation by liganded VDR occur on the opened chromatin, mediating Mediator complex with chromatin modifying enzymes. Gene regulation coupled with chromatin reorganization is considered to require chromatin remodeling complexes [9,26,27]. It is notable that not all of the target genes by liganded VDR are activated and the others are repressed [27,28]. This view reflects process and factors only for gene activation by liganded VDR, but not for transcriptional repression of the certain set of VDR target genes.

The vitamin D receptor (VDR) is a ligand-activated transcription factor belonging to the nuclear steroid/thyroid hormone receptor superfamily [11,12,13]. It mediates the biological effects of vitamin D by regulating gene expression in a ligand-dependent manner. In its inactive state, VDR resides in the cytoplasm; upon binding its ligand (the active form of vitamin D), it undergoes a conformational change, heterodimerizes with the retinoid X receptor (RXR), and is translocated to the nucleus [11,14,15]. The VDR–RXR complex binds to vitamin D response-elements (VDREs) in target gene promoters, recruiting coactivators or corepressors to modulate transcription [12,16] (Figure 1). Through this mechanism, VDR influences a broad array of physiological processes including calcium/phosphate homeostasis, bone remodeling, cellular proliferation and differentiation, immune modulation, and even neuromuscular and mood regulation [12,17]. VDR is expressed in most human tissues, with particularly high levels in classical targets such as intestine, kidney, and bone [11,18]. Notably, VDR activation can directly or indirectly regulate hundreds of genes in a cell-type-specific manner (estimates range from 200 to over 1000 genes depending on the cell), underscoring its pivotal role as a genomic regulator [19,20]. Regarding the diverse biological actions of vitamin D, the genes encode proteins related with mineral metabolism, cell–cell adhesion, proliferation, tight junction, cell proliferation, immune response, and energy consumption [21,22].

### 2.1. Ligand-Dependent Structural Alterations of the VDR Protein

The vitamin D receptor (VDR) comprises several functional domains, among which two core domains are indispensable for ligand-dependent transcriptional regulation. The central region of VDR contains the DNA-binding domain (DBD), whereas the ligand-binding domain (LBD) resides at the C-terminal end. The LBD consists of 12 α-helices (H1–H12) forming a hydrophobic pocket that accommodates ligands [23,24,29,30].

**Figure 2:**
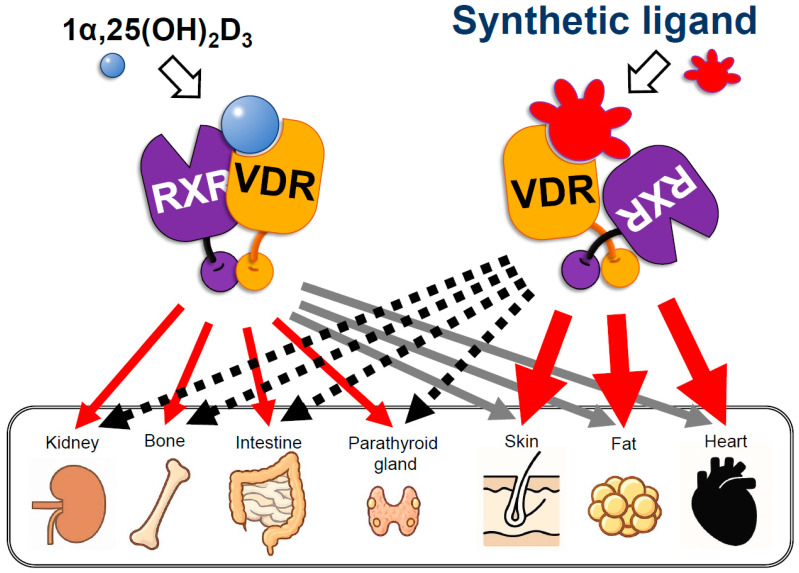
A schematic view of the tissue-specific action of a synthetic vitamin D analog. The protein structure of VDR bound a synthetic vitamin D analog can be differentially altered from VDR bound to 1α,25(OH)_2_D_3_ [4,6,31,32] and becomes potent in accommodating a particular set of transcriptional coregulators [33]. Since combination of recruited transcriptional coregulators is dependent on VDR protein structure (see Figure 1) and underlies tissue-specific function of VDR [25,26], the development of a vitamin D analog is theoretically possible. However, the tissue-specific combination of transcriptional coregulators facilitating gene regulation by VDR remains elusive.

Ligand engagement induces a pronounced conformational rearrangement, especially in helix 12 (H12). In the unliganded state, VDR associates with transcriptional corepressors, maintaining the receptor in a transcriptionally silent configuration. Upon ligand binding, H12 undergoes a substantial positional shift, causing the release of corepressors and promoting the recruitment of coactivators. This transition is accompanied by an overall structural reorganization of the VDR protein [25,26] (Figure 2).

The ligand-induced structural changes in VDR mirror those observed in other nuclear receptors (NRs) that bind endogenous ligands. Importantly, the angular displacement of H12 varies depending on the specific ligand [31,32,34]. The first compelling evidence for ligand-dependent H12 repositioning came from the crystal structure of ERα bound to raloxifene, a selective estrogen receptor modulator (SERM) [35]. Compared to the ERα–estradiol complex, the raloxifene-bound form exhibited a markedly different H12 orientation, suggesting alternative coregulator recruitment patterns that underlie raloxifene’s tissue-selective pharmacology, particularly its beneficial effects on bone [33,36].

Analogously, synthetic VDR ligands have been shown to induce distinct H12 shifts and unique VDR conformational states compared with the natural ligand 1,25(OH)_2_D_3_. These structural differences support the concept that certain synthetic VDR ligands may function as tissue-selective VDR modulators, paralleling the SERM paradigm [31,32,37] (Figure 2). The concept of ligand-dependent functional selectivity observed for VDR thus looks consistent with a broader paradigm established across the nuclear receptor superfamily. In estrogen receptor (ER) signaling, selective estrogen receptor modulators (SERMs) such as raloxifene or tamoxifen induce distinct helix-12 orientations and coregulator recruitment profiles, resulting in tissue-specific agonist or antagonist activities. Similarly, selective PPARγ modulators (SPPARMs) and glucocorticoid receptor (GR) ligands have been shown to dissociate metabolic or anti-inflammatory gene programs from adverse effects by preferential engagement of specific coactivator or corepressor assemblies, despite maintaining an overall agonist-like receptor conformation [38,39]. Comparable principles have also been reported for bile acid receptors (FXR), where endogenous bile acids and synthetic agonists elicit divergent transcriptional outputs through differential coregulator usage [40]. In this context, biased signaling of VDR ligands can be viewed as part of a conserved nuclear receptor regulatory strategy, whereby subtle ligand-induced conformational and dynamic differences—beyond the canonical helix-12 switch—fine-tune coregulator selection and downstream gene networks. Such a framework provides a mechanistic basis for understanding how synthetic VDR ligands achieve tissue- and pathway-selective actions with reduced calcemic liability (Figure 2).

The natural endocrine ligand of VDR is 1α,25-dihydroxyvitamin D_3_, also known as calcitriol, which is the hormonally active form of vitamin D_3_ [11]. Vitamin D_3_ (cholecalciferol) is synthesized in the skin under UVB radiation or obtained from dietary sources, then converted in the liver to 25-hydroxyvitamin D_3_ (calcidiol), and finally hydroxylated in the kidney to 1,25(OH)_2_D_3_ [41,42]. 1,25(OH)_2_D_3_ binds to the ligand-binding domain of VDR with high affinity (nanomolar range), inducing a receptor conformation that enables coactivator binding and gene transcription [11,43]. Key amino acid contacts in the VDR ligand-binding pocket (LBP) include interactions with residues such as Arg274, Ser278, His305, His397, etc., which stabilize 1,25(OH)_2_D_3_ within the pocket [12]. In addition to 1,25(OH)_2_D_3_, other endogenous molecules can weakly bind VDR—for example, certain bile acids (lithocholic acid) and metabolites like 20-hydroxyvitamin D_3_—but these are much less potent than 1,25(OH)_2_D_3_ and are generally considered of minor physiological relevance compared to 1,25(OH)_2_D_3_ [44,45].

### 2.2. Physiological Role of Natural VDR Ligands

1,25(OH)_2_D_3_ (1α,25-dihydroxyvitamin D_3_) is classically critical for maintaining calcium and phosphorus balance by promoting intestinal calcium absorption and bone mineralization [46]. In deficiency, bone diseases such as rickets (in children) or osteomalacia (in adults) occur [47,48]. However, over the past decades, research has revealed non-classical actions of vitamin D: it influences cell growth and differentiation, immune responses, and may have protective roles in various tissues [46,49]. Sufficient vitamin D levels have been linked to reduced risks of cancers, autoimmune disorders, and infectious diseases in epidemiological studies [49]. Consequently, there has been great interest in exploiting VDR activation therapeutically beyond bone health—for example using calcitriol or analogs to induce cancer-cell differentiation or to modulate inflammation. Indeed, vitamin D can trigger cell-cycle arrest (G0/G1 phase), promote apoptosis, inhibit angiogenesis, and modulate immunity in various cell types [50]. However, a major limitation of using calcitriol systemically as a drug is its potent calcemic activity—effective antitumor or immunomodulatory doses can cause hypercalcemia and hypercalciuria leading to kidney stones, vascular calcification, and other adverse effects [49,51]. This narrow therapeutic window has motivated the development of synthetic VDR ligands with modified structures aimed at dissociating the desirable antiproliferative or anti-inflammatory effects from the unwanted calcemic effects.

## 3. Structural Innovations and Mechanisms of Novel VDR Ligands

**Figure 3:**
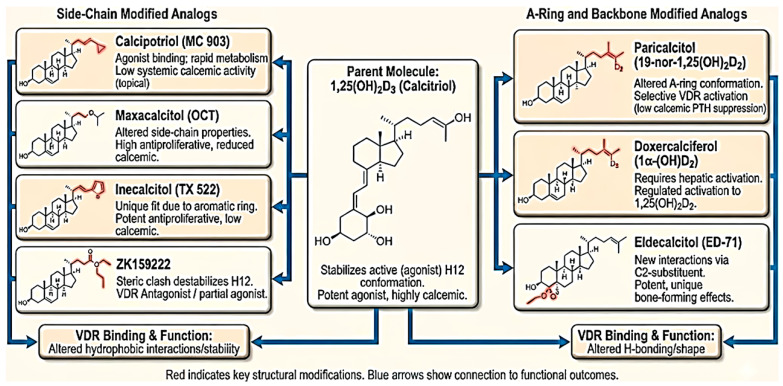
Structural comparison of 1,25(OH)_2_D_3_ and key synthetic analogs: structure–activity relationships for VDR binding. The chemical development of selected synthetic analogs and their biological activities is illustrated. Details of each compound are provided in the text.

To address this, scientists have created numerous vitamin D analogs (also called deltanoids) [27]. These analogs retain affinity for VDR but carry structural modifications (in the secosteroid core or side chain) that alter their activity profile. Several VDR analogs discussed in the article are representatively shown in Table 1. The chemical modifications are illustrated in Figure 3 to develop the analogs. For example, calcipotriol, a synthetic analog of 1,25(OH)_2_D_3_, shows markedly reduced calcium-mobilizing activity compared to 1,25(OH)_2_D_3_ yet effectively regulates keratinocyte growth and differentiation, making it an approved treatment for psoriasis [52]. Similarly, analogs like paricalcitol and doxercalciferol are used to treat secondary hyperparathyroidism (SHPT) in chronic kidney disease by suppressing parathyroid hormone with reduced hypercalcemia risk [53,54,55]. These successes illustrate that strategic chemical modifications to vitamin D can yield selective VDR modulators with improved safety profiles.

Another representative example in SHPT therapy is maxacalcitol (22-oxacalcitriol: OCT), a 1,25(OH)_2_D_3_ analog in which an oxygen atom is introduced at C-22, modifying side-chain chemistry and tissue responses relative to the natural hormone. Unlike 1,25(OH)_2_D_3_, OCT provides potent VDR-mediated suppression of parathyroid hormone in patients with chronic kidney disease on dialysis while producing less increase in serum calcium and phosphate at therapeutically equivalent doses, making it particularly suitable for managing secondary hyperparathyroidism in CKD [61,62].

In contrast, eldecalcitol (Edirol) is a 1α,25-dihydroxyvitamin D3 analog bearing a distinctive 2β-(3-hydroxypropoxy) substitution that lowers its intrinsic VDR binding affinity compared with calcitriol but markedly enhances binding to vitamin D-binding protein, prolonging its circulation time [63]. This altered pharmacology translates into a strong, sustained anti-resorptive effect on bone and superior gains in bone mineral density and fracture prevention versus older vitamin D preparations, supporting its clinical use primarily in osteoporosis rather than SHPT [64,65].

Strategic chemical modifications have successfully uncoupled some of 1,25(OH)_2_D_3_′s calcemic activities from its gene-regulatory functions. For example, removal of the C-19 methylene group, yielding so-called 19-nor analogs such as paricalcitol, results in analogs with significantly reduced calcemic effects yet retaining potent VDR-mediated transcriptional activity [66]. Additional modifications, such as epimerization at C-20 or C-21 (e.g., in 14-epi analogs), and fluorine substitution in the side chain further increase VDR potency and metabolic stability while lowering calcium mobilization [11,67].

Many of these analogs act as VDR super-agonists, exhibiting substantially enhanced receptor activation in vitro—manifested as stronger coactivator recruitment and more robust VDR–RXR dimerization—and exert antiproliferative effects at concentrations 10–100-fold lower than 1,25(OH)_2_D_3_. Classic examples include inecalcitol (TX522), a 14-epi analog that is approximately 100-fold less hypercalcemic yet more potent in inducing cancer cell differentiation and apoptosis. This heightened activity is attributed, in part, to inecalcitol’s ability to induce a tighter VDR–agonist–antagonist relationship, amplifying transcriptional regulation of antiproliferative genes [11,15].

A complementary innovative strategy involves the design of non-secosteroidal VDR ligands or VDR mimetics—structurally distinct scaffolds that fit into the VDR ligand-binding pocket via unique interactions. These compounds often induce alternative receptor conformations, sometimes bypassing classical VDR–coactivator arrangements related to calcemic outcomes, thereby enabling the development of tissue-selective VDR modulators with fewer side effects [11].

One notable example is the novel analog KK-052, derived from the vitamin D scaffold but engineered to selectively suppress SREBP-driven lipid synthesis in the liver without activating canonical VDR transcriptional programs or increasing serum calcium, demonstrating the growing capacity to dissociate metabolic and genomic VDR activities for therapeutic gains [11].

Recent crystallographic studies and molecular dynamics simulations reveal the structural basis for these effects, showing that substitutions like the C-2 side chain hydroxyl or alkyl groups enhance binding affinity and receptor activation through stabilization of the ligand-binding domain and helix 12. Moreover, partial agonism observed with specific alkyl-substituted ligands arises from mixed antagonist/agonist ligand conformers in the same crystal structure, illustrating mechanistic nuances of VDR modulation [11,41,67]

Interestingly, not all synthetic vitamin D receptor (VDR) ligands act solely as agonists—many classic agonists can elevate blood calcium by activating vitamin D-responsive genes. Some newer compounds are designed to function as VDR antagonists or partial agonists, which enable more precise control of VDR signaling [51]. These antagonistic ligands represent a promising strategy, as they may preserve positive effects of vitamin D in specific tissues while markedly lowering the risk of hypercalcemia, thanks to the tissue- and cell-type-selective actions of the VDR–RXR heterodimer [27]. This concept echoes the function of selective estrogen receptor modulators (SERMs) like raloxifene, which can behave as antagonists or agonists depending on the organ or cell type involved [33,68]. Structural studies using X-ray crystallography show that compounds like SERMs can reshape the ligand-binding domain of receptors such as ERα, thereby modifying coregulator interactions and producing distinctive gene expression patterns across tissues [35].

Recent research has characterized two distinct molecular mechanisms employed by carboxylic ester analogs to achieve VDR antagonism: coactivator exclusion (exemplified by ZK159222) and cytosolic sequestration (exemplified by ZK168281).

ZK159222, a 25-carboxylic ester analog of 1α,25(OH)_2_D_3_, was characterized as a novel VDR antagonist with a partial agonistic character. Its mechanism is primarily based on a lack of ligand-induced interaction of the VDR with coactivators. ZK159222 demonstrates high binding affinity for VDR, comparable to the natural hormone (with Kd values of 0.61 nM and 0.45 nM, respectively). This high affinity allows it to effectively compete for the ligand-binding cleft. However, once bound, ZK159222 is unable to promote a ligand-dependent interaction with coactivator proteins of the p160 family, specifically SRC-1, TIF2, and RAC3. This lack of interaction is thought to result from ZK159222 stabilizing an alternative VDR conformation that functionally blocks VDR’s transactivation function 2 (AF-2) domain located in helix 12. Although antagonism was demonstrated in vivo (in cell culture assays), a 100-fold higher concentration of ZK159222 compared to 1α,25(OH)_2_D_3_ was required to achieve a prominent antagonistic effect, reflecting its relative reduction in functional potency [69].

In contrast, the more recently studied analog ZK168281 (ZK) achieves antagonism through a mechanism independent of agonist–antagonist interaction and involves cytosolic sequestration. ZK functions by enhancing the interaction between the VDR and WBP4 (also known as FBP21), a newly identified cytosolic VDR interactant. This enhanced VDR/WBP4 interaction anchors VDR within the cytosol, thereby impairing its nuclear translocation induced by 1α,25(OH)_2_D_3_. Crucially, while ZK was initially thought to have mixed activities, it is now shown to be a potent VDR antagonist, with any associated agonistic activities attributable solely to its CYP24A1-generated derivatives (metabolites). Due to this unique mechanism, ZK has demonstrated potent and safe therapeutic efficacy in vivo, normalizing both hypercalcemia and VDR target gene expression in 1α,25(OH)_2_D_3_-intoxicated mice, making it a promising strategy for treating vitamin D-induced hypercalcemia [32].

Following the carboxylic ester analogs, DLAM-2b, a derivative of the synthetic 1α,25-dihydroxyvitamin D_3_-26,23-lactams (DLAM-2s), was developed by a group of researchers in Japan and published in late 2023. In the study by Iwaki et al., DLAM-2a–d (the “DLAM-2” series) were synthesized as 1α,25-dihydroxyvitamin D_3_-26,23-lactams that bind human VDR in competitive assays and showed antagonistic effects on 1,25(OH)_2_D_3_-induced differentiation in HL60 cells [28]. In luciferase reporter assays in COS-1 cells with human VDR, DLAM-2b acted as a transcriptional antagonist of the VDR’s activation by 1α,25(OH)_2_D_3_. It inhibited endogenous expression of the well-known vitamin D target gene CYP24A1 in human keratinocytes (HaCaT) treated with 1α,25(OH)_2_D_3_. Crucially, genome-wide RNA-seq showed that DLAM-2b regulates a distinct set of genes compared to 1,25(OH)_2_D_3_: among upregulated genes under 1,25(OH)_2_D_3_ (~246 genes) and under DLAM-2b (~163 genes), only 24 were overlapping; for downregulated genes, likewise there was only low overlap. Moreover, ATAC-seq analyses indicated that while DLAM-2b binds VDR and engages a VDRE, it did not produce the same chromatin remodeling effects (in HaCaT cells) as 1,25(OH)_2_D_3_, implying a different conformational/coregulator recruitment profile [28].

Structurally, DLAM-2 analogs were designed with a lactam in the side-chain (26,23-lactam) to reduce calcemic activity and shift VDR modulation to antagonist/partial agonist mode [70]. Although immunomodulatory effects per se in immune cells of DLAM-2b are not yet reported in vivo, the distinct gene-regulatory profile suggests that DLAM-2b (and by extension other VDR partial antagonists) could allow selective suppression or modulation of VDR-mediated immune/inflammatory genes, while avoiding induction of classical VDR target pathways that mediate calcium absorption.

In practical terms, this means that DLAM-2b represents a tool compound and potential lead for designing VDR-targeted immunomodulatory therapies that avoid hypercalcemia. It opens the possibility of biased VDR ligands—compounds that shift VDR signaling from its classical mineral metabolism mode toward immunomodulatory/anti-inflammatory modes or even selective suppression of pro-inflammatory VDR programs. This is analogous to selective modulators in other nuclear receptor families (e.g., SERMs for ER, selective GR modulators) where ligand structure can bias coregulator recruitment and downstream gene networks.

Given the known role of VDR in immune homeostasis, macrophage/dendritic cell activation, T-cell polarization and cytokine regulation, the ability to custom-tune VDR ligand activity (agonist vs. antagonist vs. partial agonist) holds substantial promise. DLAM-2b is a prime example of how synthetic ligand design has progressed from “enhanced agonists” toward functionally selective modulators of VDR signaling.

## 4. The Role of the Vitamin D Receptor in Various Pathological Conditions and Novel Directions for the Development and Application of Synthetic Ligands

### 4.1. The Vitamin D Receptor in Tumor Contexts

The vitamin D receptor (VDR) plays a vital role in regulating hundreds of genes governing cell proliferation, apoptosis, differentiation, and immune function [71,72]. Over the past two decades, evidence has revealed that VDR signaling undergoes extensive dysregulation in cancer. These alterations span ligand metabolism, epigenetic silencing, chromatin remodeling, genetic polymorphisms, and post-translational modifications [20,72]. Understanding these multifaceted changes is essential for refining vitamin D-based interventions and designing novel therapeutic strategies.

#### 4.1.1. Altered Ligand Metabolism: The CYP24A1 Axis

Vitamin D metabolism critically determines VDR activity. CYP27B1 catalyzes the conversion of 25-hydroxyvitamin D to active 1,25-dihydroxyvitamin D, whereas CYP24A1 catabolizes 1,25(OH)_2_D_3_ (and 25-hydroxyvitamin D), thereby constraining VDR signaling. In multiple cancers—including colorectal, breast, ovarian, and lung—frequent dysregulation is observed, typically *CYP24A1* upregulation and/or *CYP27B1* downregulation, which can functionally diminish VDR signaling within the tumor microenvironment [20,46,73,74]. Clinical evidence shows that *CYP24A1* overexpression is associated with poor prognosis across cancers. A meta-analysis of 3784 patients found that high *CYP24A1* correlated with worse overall survival (HR ~1.21), greater metastasis (OR ~1.81), and increased recurrence (OR ~2.14) [75]. In lung adenocarcinoma, high tumor *CYP24A1* mRNA levels predicted reduced survival [76]. In ovarian carcinoma, elevated *CYP24A1* expression was observed in tumor versus adjacent tissues, and this metabolic change skewed macrophage polarization toward tumor-promoting M2 states [77]. Experimental xenograft models further confirmed that *CYP24A1* overexpression accelerates colorectal tumor growth and proliferation [78].

#### 4.1.2. Non-Classical VDR Activation Pathways: Ligand Generation by CYP11A

**Figure 4:**
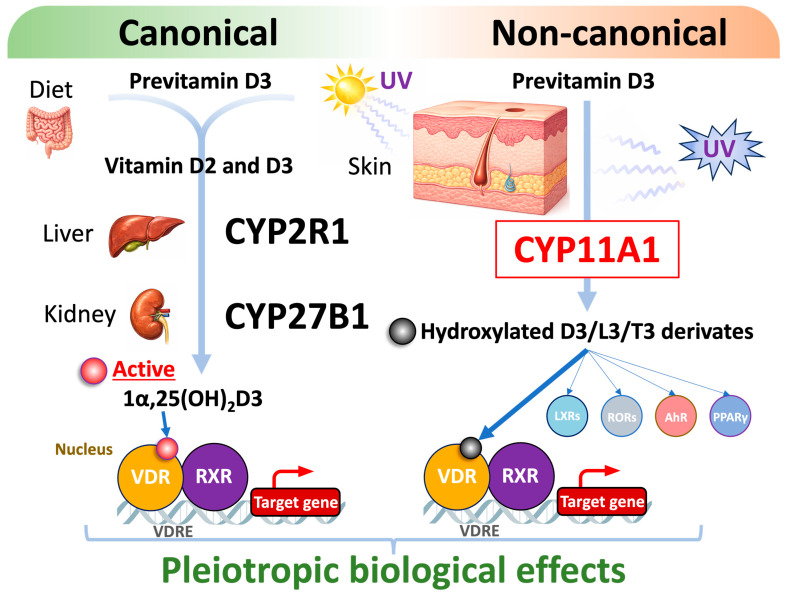
Non-canonical vitamin D activation pathways. The active form of vitamin D that serves as a ligand for the VDR has classically been attributed to 1,25-dihydroxyvitamin D_3_ (1,25(OH)_2_D_3_) and its derivatives. However, recent studies have revealed the presence of non-classical ligand-generation pathways mediated by CYP11A1 and UV exposure [79,80,81]. Lumisterol and vitamin D hydroxyderivatives generated by CYP11A1, in cooperation with other cytochrome P450 enzymes, as well as UV exposure of the skin have been reported to act as ligands not only for VDR but also for other members of the nuclear receptor family.

VDR activation has classically been attributed to 1,25-dihydroxyvitamin D_3_ (1,25(OH)_2_D_3_) and closely related metabolites. However, recent studies have identified alternative, non-classical ligand-generation pathways mediated by CYP11A1 (Figure 4) [79,80,81].

CYP11A1, together with cooperating cytochrome P450 enzymes, catalyzes the conversion of vitamin D_3_ and lumisterol into a series of hydroxylated secosteroids, collectively referred to as lumisterol and vitamin D hydroxyderivatives. These metabolites function as biologically active ligands not only for VDR but also for other nuclear receptors, including liver X receptors (LXRs) and retinoic acid-related orphan receptors (RORs), as well as the aryl hydrocarbon receptor (AhR) [79,80,81]. Unlike calcitriol, many of these CYP11A1-derived ligands exhibit non-calcemic activity and act through partial agonism or inverse agonism, thereby modulating VDR, RORγ, PPARγ, and AhR signaling. It is also notable that such NR ligands and related cholesterol derivatives are also generated in the skin by UV [82]. Through this multi-receptor engagement, they reprogram transcriptional networks governing cellular differentiation, immune regulation, metabolism, and epithelial homeostasis. These findings indicate that endogenous VDR ligands generated by CYP11A1 are not strictly VDR-selective but instead coordinately regulate multiple receptor pathways, revealing an additional layer of complexity in vitamin D signaling and suggesting new opportunities for therapeutic intervention based on biased or multi-receptor ligand action.

#### 4.1.3. Epigenetic Regulation of VDR

Beyond ligand metabolism, epigenetic mechanisms contribute to VDR inactivation. In colorectal, breast, and cervical cancers, hypermethylation of the VDR promoter has been observed, reducing transcription and protein levels. For instance, in breast cancer, hypermethylation of about 700 base pairs upstream as well as close to the transcription start site of the VDR gene has been demonstrated and linked to reduced VDR expression and VDRE-containing genes such C/EBP and p21 [78]. In cancerous colorectal tissue, the frequency of VDR promotor methylation was about four times higher than in normal tissue, and methylation status was significantly associated with tumor staging [83].

1,25(OH)_2_D_3_ itself participates in epigenetic crosstalk: it modulates DNMT and HDAC expression, alters histone-acetylation marks such as H3K27ac, and regulates lncRNAs like *HOTAIR* and *H19*, which interact with chromatin modifiers of VDR targets [84,85]. Consequently, combined use of vitamin D analogs with epigenetic drugs (e.g., DNMT or HDAC inhibitors) shows promise for reactivating silenced VDR pathways in resistant tumors.

A third layer of control comes from oncogene-driven and EMT-associated transcriptional repressors. Transformation studies demonstrate that oncogenes such as *SV40* and *RAS* reduce VDR expression and activity by more than 70% compared to non-transformed mammary cells [86]. More prominently, members of the SNAIL family (SNAIL1/SNAI1 and SLUG/SNAI2) directly repress VDR transcription by binding to E-box motifs within its proximal promoter. SNAIL1 also destabilizes VDR mRNA, while combined expression of SNAIL1 and SNAIL2 exerts additive suppression, abolishing the antiproliferative effects of 1,25(OH)_2_D_3_ [87]. The downregulation of VDR by SNAIL family transcription factors is a conserved oncogenic mechanism not confined to colorectal cancer (CRC). Empirical evidence demonstrates that SNAIL1 and SNAIL2 suppress VDR expression and consequently abrogate the antitumor response to 1,25(OH)_2_D_3_ in human osteosarcoma and breast cancer models, respectively [88,89]. Given the widespread overexpression of SNAIL proteins in cancer, this pathway likely explains, at least in part, the consistently reduced VDR levels documented in melanomas, breast, lung, and ovarian tumors [90,91,92]. Beyond chromatin and transcriptional control, noncoding RNAs provide a crucial post-transcriptional regulatory layer [93]. MicroRNAs (miRNAs) can directly target the VDR 3′UTR, leading to reduced receptor stability and translation [94]. For instance, *miR-125b* suppresses VDR in breast cancer cells, although its downregulation in some tumors may paradoxically allow VDR upregulation [95]. In contrast, *miR-214* and *miR-1204* repress VDR in breast cancer cell lines, attenuating 1,25(OH)_2_D_3_ responsiveness [96,97]. Moreover, vitamin D itself regulates miRNAs such as *miR-145*, which is induced by 1,25(OH)_2_D_3_ in gastric cancer cells and mediates antiproliferative effects through targeting E2F3 [98]. This bidirectional relationship indicates that vitamin D–VDR signaling is not only subject to miRNA regulation but also actively reshapes the noncoding RNA landscape.

#### 4.1.4. Chromatin Reprogramming and the VDR Cistrome

VDR function depends not only on expression but also on its cistrome, the set of genomic binding sites accessible in a given cell type. Tumors can reprogram this cistrome, reshaping the transcriptional output of VDR signaling. High-resolution ATAC-seq and ChIP-seq studies in prostate cancer have demonstrated that 1,25(OH)_2_D_3_ induces cell-type-specific shifts in chromatin accessibility and VDR binding. Notably, African American prostate cancer cells showed a quantitatively distinct VDR cistrome compared to European American cells, partly due to reduced expression of the chromatin remodeler BAZ1A. Restoration of BAZ1A rescued some VDR-dependent chromatin accessibility and transcriptional responses; see Siddappa et al., 2023 [99]. This evidence underscores the importance of chromatin context. Even if VDR protein is present and its ligand is available, inaccessible chromatin can prevent VDR from engaging its canonical tumor-suppressive targets. The parallels with androgen receptor cistrome reprogramming in prostate cancer suggest a general principle for nuclear receptor signaling in malignancy [100].

#### 4.1.5. Genetic Polymorphisms and Isoforms

Genetic variation in the VDR pathway modulates cancer susceptibility and outcomes. Several polymorphisms (FokI, BsmI, ApaI, and TaqI) have been widely studied, with variable associations depending on cancer type and ethnicity. The FokI polymorphism alters the translational start site, producing isoforms of different length and transcriptional activity [101], and was reported to be associated with inflammatory skin disease such as psoriasis and atopic dermatitis [102,103]. In addition, *CYP24A1* polymorphisms have been associated with altered cancer risk and therapy response. A meta-analysis reported significant associations between *CYP24A1* SNPs (e.g., rs4809960, rs2296241) and breast, prostate, and esophageal cancer susceptibility [104]. These findings highlight the importance of integrating host genetics into studies of vitamin D responsiveness and patient stratification for clinical interventions.

Despite extensive promising preclinical data, it must be noted that no VDR analog is yet an established anticancer drug in routine clinical practice [51,105]. Several factors contribute to this: (1) difficulty in dosing sufficiently high to see efficacy without side effects; (2) cancer cells developing resistance (e.g., by upregulating CYP24A1 enzyme that degrades 1,25(OH)_2_D_3_, or downregulating VDR itself) nature.com; (3) the complexity of cancer biology where single-agent vitamin D may be insufficient [49,51]. Nonetheless, ongoing clinical trials are investigating VDR ligands in combination with other therapies (e.g., with anti-estrogens in breast cancer, with immunotherapy in melanoma/GBM, with chemotherapy in pancreas and colon cancer). The consensus in the field is that vitamin D by itself might not cure advanced cancers, but synthetic VDR ligands could be valuable as part of multi-modal therapy or in chemoprevention. They are generally well-tolerated in patients at moderate doses, and even the hypercalcemia risk can be managed with careful monitoring or intermittent dosing schedules. As safer analogs (e.g., second- and third-generation deltanoids like MART-10, inecalcitol, or newer non-secosteroidal compounds) are developed, the prospect of incorporating VDR-based therapies in oncology grows. Indeed, the ability of VDR activation to simultaneously target cancer cell growth, differentiation, and the immune microenvironment is unique and highly appealing for future cancer therapeutics [11,106].

### 4.2. Metabolic Disease and Anti-Fibrotic Applications

VDR signaling also intersects with metabolic and fibrotic diseases, and synthetic ligands are showing therapeutic potential in these domains. In the context of non-alcoholic fatty liver disease (NAFLD) and metabolic syndrome, VDR activation in immune cells can ameliorate chronic inflammation and insulin resistance. A 2020 study demonstrated that treating obese mice with the VDR agonist calcipotriol significantly reduced liver inflammation and steatosis (fatty liver), while improving insulin sensitivity in vivo [56]. The benefits were traced to VDR activation in liver macrophages (Kupffer cells), which dampened pro-inflammatory cytokine release and protected hepatic insulin signaling. Notably, mice lacking VDR in these cells developed worse metabolic inflammation, underscoring VDR as a therapeutic target in NASH/NAFLD [56]. This proof-of-concept suggests that vitamin D analogs could be repurposed for metabolic liver diseases, an idea now being explored in clinical trials (e.g., paricalcitol is under investigation for NASH fibrosis).

Another promising area is organ fibrosis, often driven by pro-fibrotic TGF-β signaling. Intriguingly, VDR can interfere with TGF-β/SMAD pathways independently of its usual genomic action, by directly binding and sequestering SMAD transcription factors [57]. Researchers have exploited this by designing ligands that favor the anti-fibrotic, non-genomic action of VDR while minimizing transcription of calcium-regulating genes. For example, 1,25D_3_-lactone (a naturally occurring metabolite) and novel synthetic lactone analogs (DLAM compounds) were found to inhibit TGF-β-induced pro-fibrotic gene expression without causing hypercalcemia [57]. Structural studies showed these lactone analogs bind VDR in an altered conformation (helix-12 positioning) compared to 1,25(OH)_2_D_3_, explaining their ability to block fibrosis signaling selectively. Such compounds effectively prevented myofibroblast activation in the kidney and skin in preclinical models. Likewise, in liver and pancreas injury models, VDR agonists (including calcipotriol and paricalcitol) have suppressed fibrogenesis: treating mice with these ligands reduced collagen deposition and activation of hepatic/pancreatic stellate cells, translating into attenuated fibrosis in chronic liver injury and pancreatitis settings. In fact, VDR-knockout mice exhibit spontaneous liver fibrosis, whereas VDR activation keeps stellate cells in a quiescent, anti-fibrotic state [57]. These findings open the door to using VDR ligands as anti-fibrotic agents in diseases like liver cirrhosis, renal fibrosis, and even desmoplastic tumors.

### 4.3. Immunomodulatory and Anti-Inflammatory Effects

The immunoregulatory properties of vitamin D are well documented—VDR activation tends to promote tolerogenic and anti-inflammatory immune responses. Recent reviews (e.g., Artusa & White 2025) emphasize that VDR agonism can shift T-cell balance (suppressing Th1/Th17 while boosting T-reg cells) and enhance antimicrobial defenses [6,57]. In autoimmune diseases such as multiple sclerosis and type 1 diabetes, vitamin D deficiency correlates with higher disease risk, and 1,25(OH)_2_D_3_ shows protective effects in animal models. However, the hypercalcemia from high-dose calcitriol precludes its chronic use in these conditions. Here, synthetic ligands known as selective VDR modulators are being explored to dissociate immunomodulatory benefits from calcemic side effects. For instance, the analog calcipotriol (already used safely in psoriasis patients) was shown to reduce inflammatory cytokine production in skin and improve psoriatic lesions via local VDR activation, with negligible systemic calcium impact [57]. Likewise, experimental VDR agonists have been reported to ameliorate inflammatory bowel disease and rheumatoid arthritis in animal studies, primarily by suppressing pro-inflammatory gene programs in macrophages and dendritic cells [57].

## 5. Conclusions

This review highlights how diversification of the vitamin D scaffold has expanded the VDR ligand space from calcitriol to selective modulators, prodrugs, and non-secosteroidal chemotypes. By biasing VDR conformation and coregulator recruitment, these ligands can dissociate antiproliferative, immunomodulatory, and anti-fibrotic actions from calcemic liability. At the same time, endogenous non-canonical ligand pathways and cell-contextual determinants such as chromatin accessibility, epigenetic repression, and noncoding RNA regulation shape VDR output in health and disease. Future translational progress will likely depend on mechanism-based ligand design with clearly defined bias/selectivity, biomarker-driven patient stratification (including VDR/CYP24A1 status and host genetics), and rational combination regimens that exploit VDR biology to remodel tumor–stroma–immune interactions while maintaining safety.

## Figures and Tables

**Table 1:** Representative natural and synthetic VDR ligands: target indications and pharmacological characteristics.

Ligand	Classification	Target Disease/Clinical Status	Key Characteristics & Mechanism of Action
Calcitriol	Natural Hormone	Approved: Rickets, osteomalacia, osteoporosis [47,48] Limitation: Systemic use in oncology is limited by toxicity [49,51]	The endogenous high-affinity ligand for VDR [11,43]. Critical for calcium/phosphate homeostasis and bone mineralization [46]. Exhibits potent antiproliferative effects but induces dose-limiting hypercalcemia [49,50,51].
Calcipotriol	Synthetic Analog (Side-chain modified)	Approved: Psoriasis [10,11] Experimental: NAFLD, Liver fibrosis [56,57]	Dissociated Activity: Markedly reduced calcium-mobilizing potential compared to calcitriol [27,52]. Potently regulates keratinocyte growth and differentiation [27,52]. Shows anti-inflammatory effects in liver macrophages [56].
Paricalcitol	Synthetic Analog (19-nor)	Approved: Secondary hyperparathyroidism (CKD) [10,11] Experimental: Pancreas [57]	Suppresses parathyroid hormone (PTH) with significantly reduced calcemic side effects [53,55]. Demonstrates anti-fibrotic activity in pancreas injury models [57].
Inecalcitol (TX522)	Synthetic Analog (14-epi)	Clinical Investigation: Advanced cancers (oncology) [58,59]	Super-agonist: Induces tighter VDR–agonist–antagonist relationships and enhanced antiproliferative signaling [15]. Approx. 100-fold lower hypercalcemic risk than calcitriol, allowing higher dosing for tumor suppression [59].
DLAM-2b	Synthetic Modulator (Lactam derivative)	Experimental: Immunomodulation, inflammatory diseases [28]	Partial Antagonist: Transcriptional antagonist for classical VDR pathways (avoiding hypercalcemia) [28]. Regulates a distinct gene set mainly associated with immune modulation rather than mineral metabolism [28].
KK-052	Non-secosteroidal (Novel scaffold)	Experimental: Metabolic diseases (e.g., fatty liver) [60]	Metabolic Selector: Selectively suppresses SREBP-driven lipid synthesis [60]. Does not activate canonical VDR genomic programs or elevate serum calcium, demonstrating dissociation of metabolic and calcemic activities [60].
Maxacalcitol (22-oxacalcitriol/OCT)	Synthetic Analog (22-oxa)	Approved: Secondary hyperparathyroidism (SHPT) (used in CKD/dialysis patients)	PTH Suppression: Effectively suppresses PTH secretion and parathyroid cell proliferation. Rapid clearance and reduced intestinal calcium absorption contribute to a lower risk of hypercalcemia compared to calcitriol.
Edirol (Eldecalcitol)	Synthetic Analog (2β-hydroxypropyloxy)	Approved: Osteoporosis	Bone Specificity: Possesses a unique mechanism that strongly inhibits bone resorption and stimulates bone formation. Superior to alfacalcidol in increasing bone mineral density and preventing fractures.

## Data Availability

No new data were created or analyzed in this study. Data sharing is not applicable to this article.
